# Daily fall risk assessment for older adults using a balance sensor with machine learning

**DOI:** 10.3389/fpubh.2026.1827730

**Published:** 2026-06-24

**Authors:** Jiaming Chen, Xin Ma, Ke Han Zou, Yuqing Tian, Qingqing Zhang, Bi Sheng, Vivian W. Q. Lou, Ning Xi

**Affiliations:** 1 Department of Data and System Engineering, The University of Hong Kong, Hong Kong, Hong Kong SAR, China; 2 Department of Social Work and Social Administration, Sau Po Centre on Ageing, The University of Hong Kong, Hong Kong, Hong Kong SAR, China

**Keywords:** balance sensor, center of gravity, center of pressure, older adults, fall assessment, machine learning

## Abstract

**Introduction:**

Fall-risk assessment is important for preventing falls in older adults, but many conventional balance tests are time-consuming and difficult to use frequently in daily practice. This study aimed to evaluate a rapid 30-s quiet-standing assessment using a balance sensor combined with machine learning for fall-history classification and fall-risk assessment.

**Methods:**

A total of 394 older adults (mean age 71.56 ± 6.69 years) participated, including 91 individuals in a laboratory cohort, 291 in an independent hospital cohort, and 12 in a 10-day home-based feasibility study. The balance sensor captured plantar pressure-distribution images during quiet standing. From these recordings, center-of-pressure (CoP) trajectories and model-derived center-of-gravity (CoG) descriptors were obtained. Spatial, stability, frequency, temporal, and statistical features were extracted, and multiple machine-learning models were evaluated. Model development in the laboratory cohort used nested stratified cross-validation, and the final pipeline was externally validated in the hospital cohort.

**Results:**

In the laboratory cohort, the highest area under the receiver operating characteristic curve (AUC) was 0.71, achieved by the random forest model under the eyes-open condition. Under the eyes-closed condition, the highest AUC was 0.68. In the independent hospital cohort, external validation of the final laboratory-developed model yielded an AUC of 0.68 and an accuracy of 72.0%, which was numerically higher than those of the conventional functional assessments evaluated in this study. The home-based component supported the feasibility of repeated short-duration balance measurement outside the laboratory.

**Discussion:**

These findings suggest that a brief sensor-based standing assessment combined with machine learning may provide a practical approach for fall-history classification and fall-risk assessment in older adults. However, the main analyses were based on retrospective fall history or documented clinical fall risk rather than prospectively observed future falls. Larger prospective and multi-center studies are needed to determine predictive validity and broader clinical utility.

## 1 Introduction

Falls are the second leading cause of accidental injury-related deaths worldwide, surpassed only by road traffic injuries, with approximately 684,000 fatalities each year, according to the World Health Organization (1). Although most falls are non-fatal, an estimated 37.3 million annually are severe enough to require medical attention (1). Fatal fall-related injuries disproportionately affect middle-aged and older adults. Among older adults, the global pooled prevalence of falls has been estimated at 26.5% (95% CI: 23.4–29.8%) (2). As population ageing continues worldwide, the health and social burden associated with falls is likely to increase further. In China, falls have become a major public health concern among older adults, and national surveillance data and related studies have shown a substantial burden of fall-related injury in this population (3, 4).

The high prevalence of falls in older populations highlights the need for effective strategies for fall-risk assessment and prevention. Maintaining postural balance is fundamental to fall prevention. However, traditional balance assessment methods, including clinical rating scales and posturographic testing, are not always well suited to frequent monitoring in daily practice. Current assessment tools, such as the Hendrich II Fall Risk Model, STRATIFY Scale, Timed Up and Go test, Tinetti Balance Scale, and Berg Balance Scale, are widely used in hospitals and rehabilitation settings (5, 6). These instruments remain clinically useful, but their ability to distinguish fallers from non-fallers or to predict future falls is not consistent across populations and settings (6–10). In clinical and physiotherapy practice, traditional balance assessments mainly categorize an individual’s performance under supervised and standardized conditions. As a result, they may not be sufficiently sensitive to short-term fluctuations in balance status that occur in everyday life.

Fall risk in older adults is not static. Balance performance may vary over relatively short periods because of fatigue, medication effects, temporary physical condition, and environmental context. However, most current assessment paradigms still rely on periodic point-in-time evaluations conducted in clinics or laboratories. These assessments provide useful snapshots of function, but they may not fully capture short-term variation in balance status during daily life. This limitation is important when the goal is not only to classify general fall susceptibility, but also to support repeated monitoring and timely intervention in real-world settings.

Most previous studies on fall-risk assessment have used wearable sensors and have reported promising results in identifying older adults at higher risk of falling (11, 12). However, wearable approaches may be limited by device adherence, user acceptance, and the need for technical support in real-world deployment. Objective sway-based assessment remains attractive because it directly quantifies postural control. Prospective evidence has shown that increased postural sway during quiet stance is associated with subsequent falls in community-dwelling older adults (13, 14). At the same time, systematic reviews of sensing technologies have highlighted the growing use of inertial sensors, pressure platforms, cameras, and other non-invasive systems, while also noting persistent challenges related to validation, interpretability, and practical implementation (15, 16). These considerations support the need for an accessible and rapid balance assessment tool that can be repeatedly applied outside highly specialized laboratory settings.

Against this background, this study aimed to evaluate a practical sensor-based framework for fall-risk assessment in older adults by integrating balance-sensor data, feature extraction, and machine-learning models. Specifically, we quantified postural balance through CoP trajectories and model-derived CoG-related descriptors derived from pressure-distribution images, identified features associated with fall status, and assessed the performance of machine-learning models for fall-history classification. We further examined the applicability of the proposed approach in laboratory, clinical, and short-term home-based settings. The overall goal was to explore whether this framework could provide a practical tool for repeated balance assessment in real-world settings.

## 2 Materials and methods

### 2.1 The balance sensor

**Figure 1:**
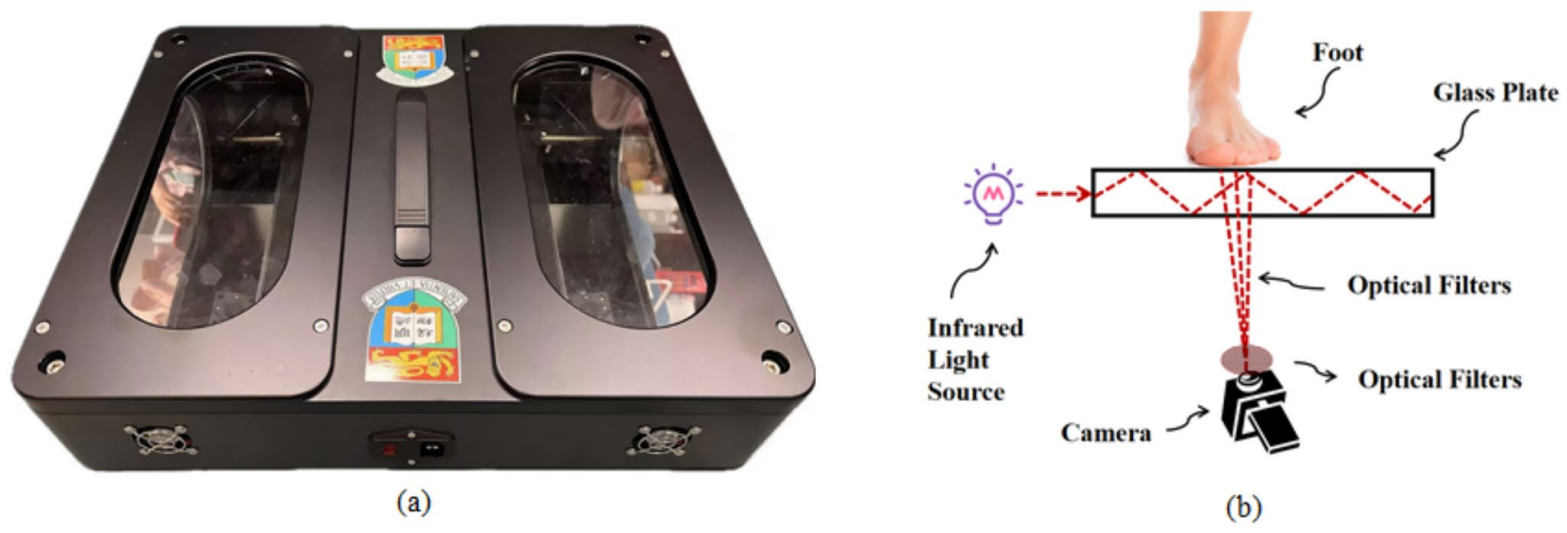
Illustration of the balance sensor system and its working principle. **(a)** Balance Sensor Hardware. **(b)** Principle of the Balance Sensor: Frustrated Total Internal Reflection, showing how contact with the glass surface disrupts light reflection.

The balance sensor employs the Frustrated Total Internal Reflection (FTIR) principle and comprises a high-resolution camera, LED light sources, and transparent glass optic-wave guides. Light emitted from the LEDs undergoes total internal reflection within the glass because of the refractive index difference between glass and air. When the plantar surface contacts the glass, this reflection is locally frustrated, producing diffused light at the contact regions that can be captured by the camera. In the present system, the recorded image intensity was used as an optical surrogate of plantar contact distribution after calibration, rather than as a direct force-platform measurement. The principle of the balance sensor is illustrated in Figure 1.

The newly developed sensor is housed in a compact black box measuring approximately 50 cm × 40 cm × 12 cm. As shown in Figure 1, the device consists of a balance-evaluation module and a computer connected via WiFi. The evaluation component contains two separate flat glass optic-wave guides and a high-resolution wireless camera, allowing the pressure-distribution patterns under the left and right feet to be captured simultaneously. When a participant stands on the glass surface, the contact areas generate diffused reflection, and the resulting haptic image represents the spatial distribution of plantar loading. The camera recorded the image stream at 30 Hz, and the data were transmitted wirelessly to the connected computer for visualization and storage.

**Figure 2:**
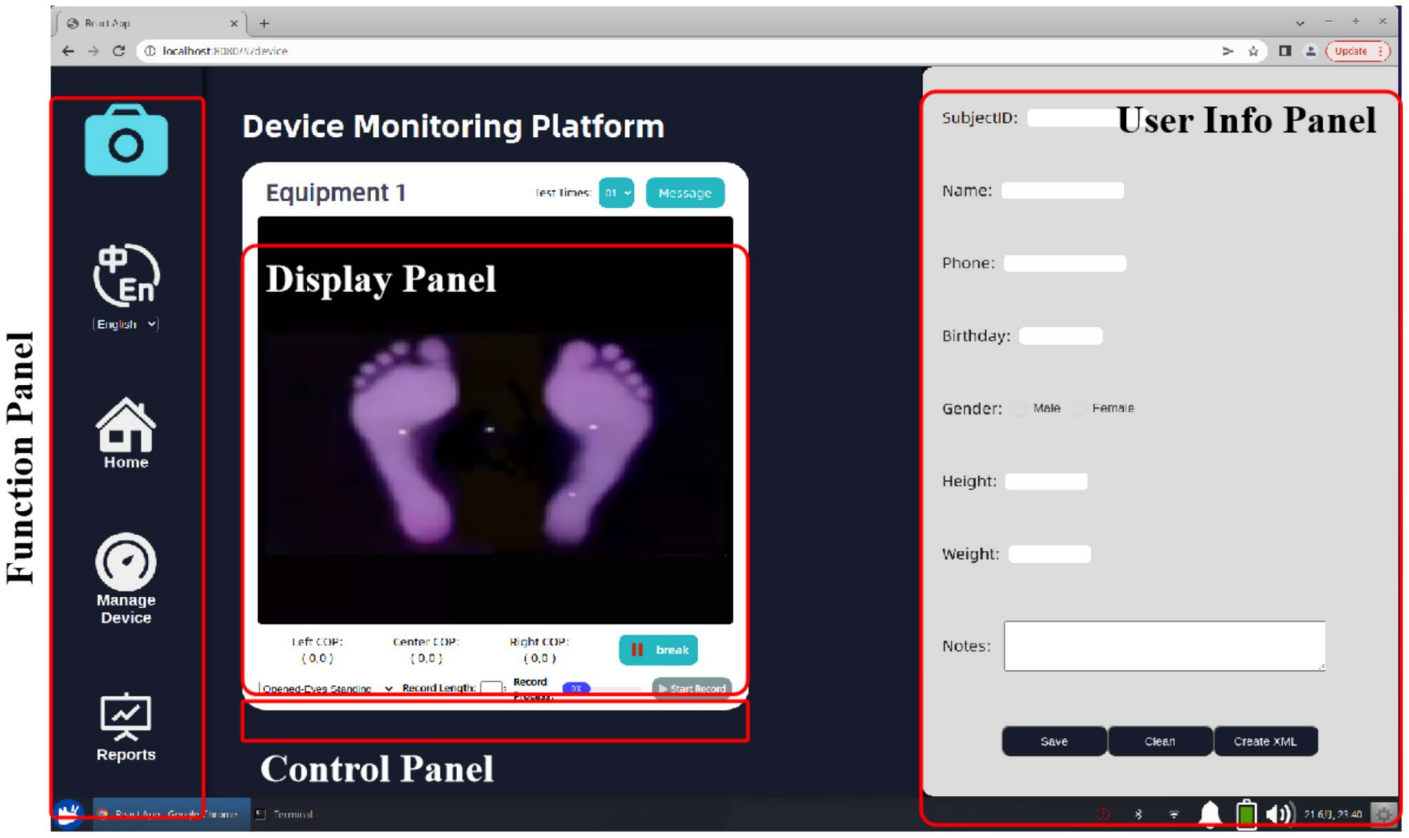
The GUI of the balance sensor system, showing the live haptic image of foot pressure distribution with highlighted centers of pressure for each foot (white dots) and the calculated center of gravity. The interface displays real-time coordinate values in the top right corner.

The graphical user interface (GUI) was developed to support data acquisition and real-time display during the balance test. As shown in Figure 2, the GUI presents the live haptic image of the participant’s feet and displays three dynamically updated points: the pressure center of the left foot, the pressure center of the right foot, and a model-derived whole-body balance point used in this study as an estimated descriptor related to CoG. These points are computed from the image using weighted averages of the relevant pixels and are displayed as white dots. The GUI was designed for measurement and visualization purposes; the final feature extraction and model analyses were performed offline after data collection.

### 2.2 Participants and protocol

Our study comprised a total of 394 participants, including 91 individuals from controlled laboratory tests, 291 participants from SYP Hospital assessments, and 12 participants from a 10-day home-based daily testing program. These three groups served different roles in the study design. The laboratory cohort was used for the main model-development analysis, the SYP Hospital cohort was used as an independent clinical validation cohort, and the 10-day home-based cohort was used only to assess the feasibility of repeated daily measurements outside the laboratory.

The average age of the study population was 71.56 ± 6.69 years, with nearly half (49.45%) having experienced at least one fall in the previous 12 months. In the main laboratory analysis, fall status was defined retrospectively based on whether a participant had experienced at least one fall during the previous 12 months. Accordingly, the present study addresses fall-history classification rather than direct prospective prediction of future falls. The majority of participants were female (78.02%), and slightly more than half (54.44%) were married. Most participants (92.31%) had received formal education, and a significant proportion (81.32%) lived with family members or caregivers rather than alone. In terms of body mass index (BMI), the largest group (46.15%) maintained a normal BMI range of 18.5–23.9, while others fell into the underweight (9.89%), overweight (25.27%), and obese (10.99%) categories.

### 2.3 Experimental protocol

The balance sensor protocol involves a series of standardized steps. Participants are instructed to remove their shoes and socks before standing on the sensor. They maintain a natural standing position for 30 s with eyes open, followed by 30 s with eyes closed. Throughout the process, one experimenter manages data collection while another adjusts the testing platform and records measurements. Participants return to a natural standing position between different movements, with rest periods included to prevent fatigue. This protocol was used to obtain short-duration quiet-standing recordings for the subsequent fall-history classification analysis in the laboratory cohort.

Additionally, we conducted three standard clinical assessments to evaluate participants’ mobility and fall risk. In the Timed Up and Go (TUG) test, participants were instructed to rise from a chair, walk 3 meters at a comfortable speed, turn around, return to the chair, and sit down, with their performance measured by completion time. For the Gait Speed Test (GST), participants walked a 4-meter distance at their normal pace, with the walking time recorded as an indicator of their functional mobility status. During the Sit-to-Stand (STS) test, participants performed five consecutive movements of standing up from and sitting back down on a chair, with the total time reflecting their lower limb strength and balance capability. These tests provide a comprehensive evaluation of motor function and are commonly used for fall risk assessment in the community and all tests were conducted under close supervision to ensure participant safety. In the home-based 10-day component, the same standing procedure was repeated to examine whether balance-sensor measurements could be practically acquired on a daily basis outside the laboratory. This part of the study was intended as a feasibility assessment and was not used to establish prospective predictive validity.

### 2.4 Data collection and feature extraction

We extracted center-of-pressure (CoP) trajectories from the pressure-distribution videos recorded by the balance sensor. All data processing and feature extraction were implemented in Python (version 3.9). To reduce transient effects at trial onset, we discarded the first 5 s of each recording as a pre-settling period. Eyes-open and eyes-closed trials were processed separately.

$$\mathrm{Co}P_{x}=\frac{\sum_{i=1}^{n}P_{i}x_{i}}{\sum_{i=1}^{n}P_{i}}$$

$$\mathrm{Co}P_{y}=\frac{\sum_{i=1}^{n}P_{i}y_{i}}{\sum_{i=1}^{n}P_{i}}$$

where $P_{i}$ denotes the pressure value at pixel $i_{1}$ , and $\left(x_{i},y_{i}\right)$ are the corresponding pixel coordinates. For each participant, the baseline CoP position was defined as the mean of ${\mathrm{CoP}}_{x}$ and ${\mathrm{CoP}}_{y}$ over all frames in the trial.

**Figure 3:**
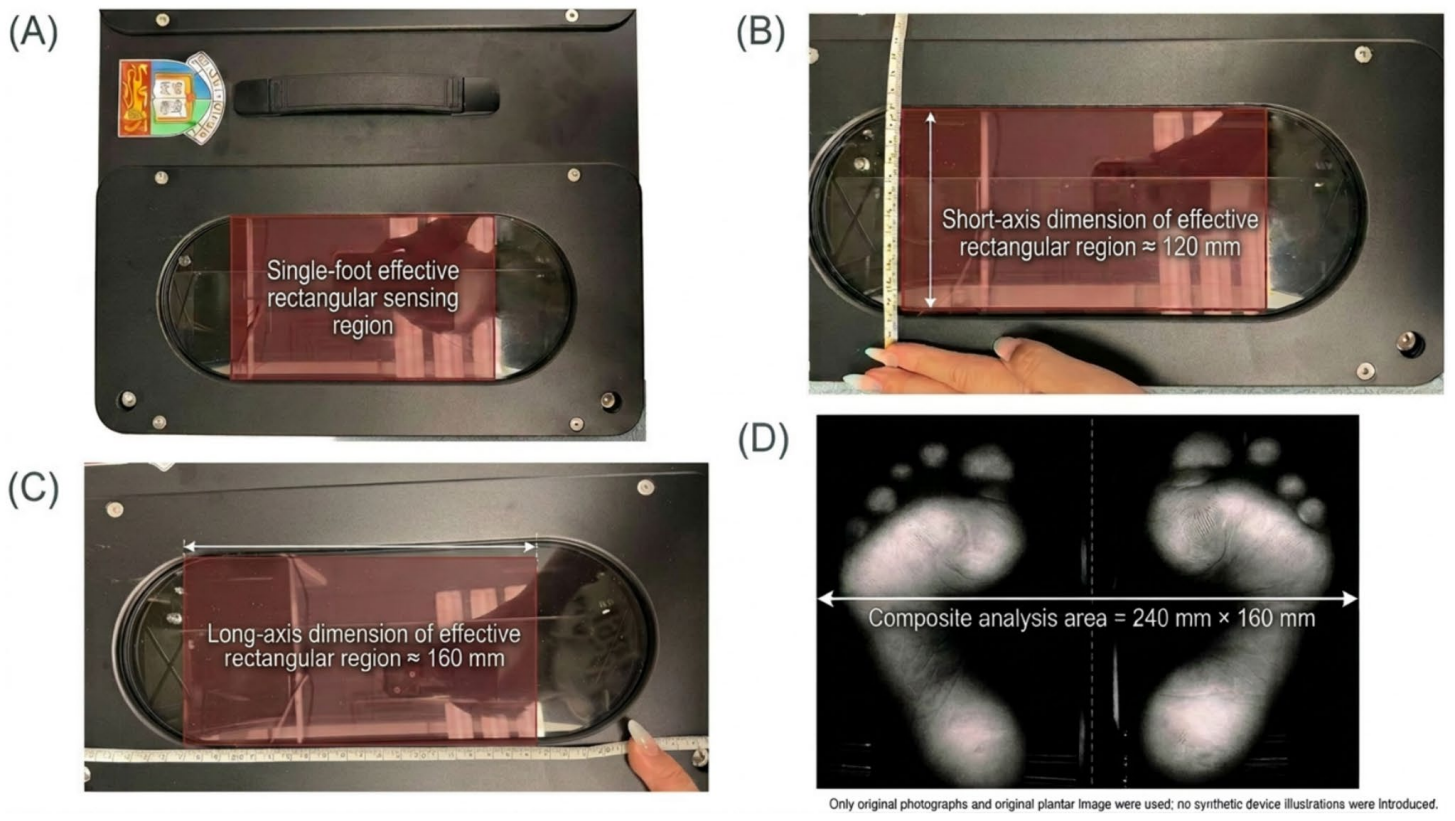
Spatial calibration procedure for pixel-to-millimeter conversion. **(A)** Photograph of the single-foot sensing window, highlighting the effective rectangular sensing region used in image analysis rather than the full outer capsule-shaped boundary. **(B)** Ruler-based measurement showing that the short-axis dimension of the effective rectangular region was approximately 120 mm. **(C)** Ruler-based measurement showing that the long-axis dimension of the effective rectangular region was approximately 160 mm. **(D)** Composite plantar image formed by horizontal merging of the left and right single-foot regions, corresponding to an effective physical analysis area of 240 mm × 160 mm. This physical analysis area was mapped to a standardized image size of 120 × 80 pixels during preprocessing, yielding a fixed spatial conversion factor of 2.0 mm/pixel in both directions.

To clarify the spatial scaling of the sensor output, all pixel-based trajectory coordinates were converted into physical coordinates using a fixed calibration factor of 2.0 mm/pixel in both the mediolateral and anteroposterior directions. This conversion factor was derived from the effective rectangular sensing region used in the image-processing pipeline rather than from the full outer dimensions of the capsule-shaped glass window. As shown in Figures 3A–C, the effective sensing region for a single foot was approximately 120 mm × 160 mm, based on ruler-supported measurements of its short-axis and long-axis dimensions. During preprocessing, the left and right single-foot plantar images were horizontally merged into one composite image (Figure 3D), corresponding to an effective physical analysis area of 240 mm × 160 mm. The composite image was then resized to a standardized resolution of 120 × 80 pixels for trajectory extraction and subsequent analysis. Because this standardized image size preserved the aspect ratio of the effective physical analysis area, the resulting spatial scale was 240/120 = 2.0 mm/pixel in the horizontal direction and 160/80 = 2.0 mm/pixel in the vertical direction. Thus, a fixed isotropic calibration factor of 2.0 mm/pixel was applied to all participants, and the converted coordinates were calculated as $x_{\mathrm{mm}}=2x_{\mathrm{px}}$ and $y_{\mathrm{mm}}=2y_{\mathrm{px}}$ . All displacement-related metrics reported in the revised manuscript were recalculated after this pixel-to-millimeter conversion.

**Figure 4:**
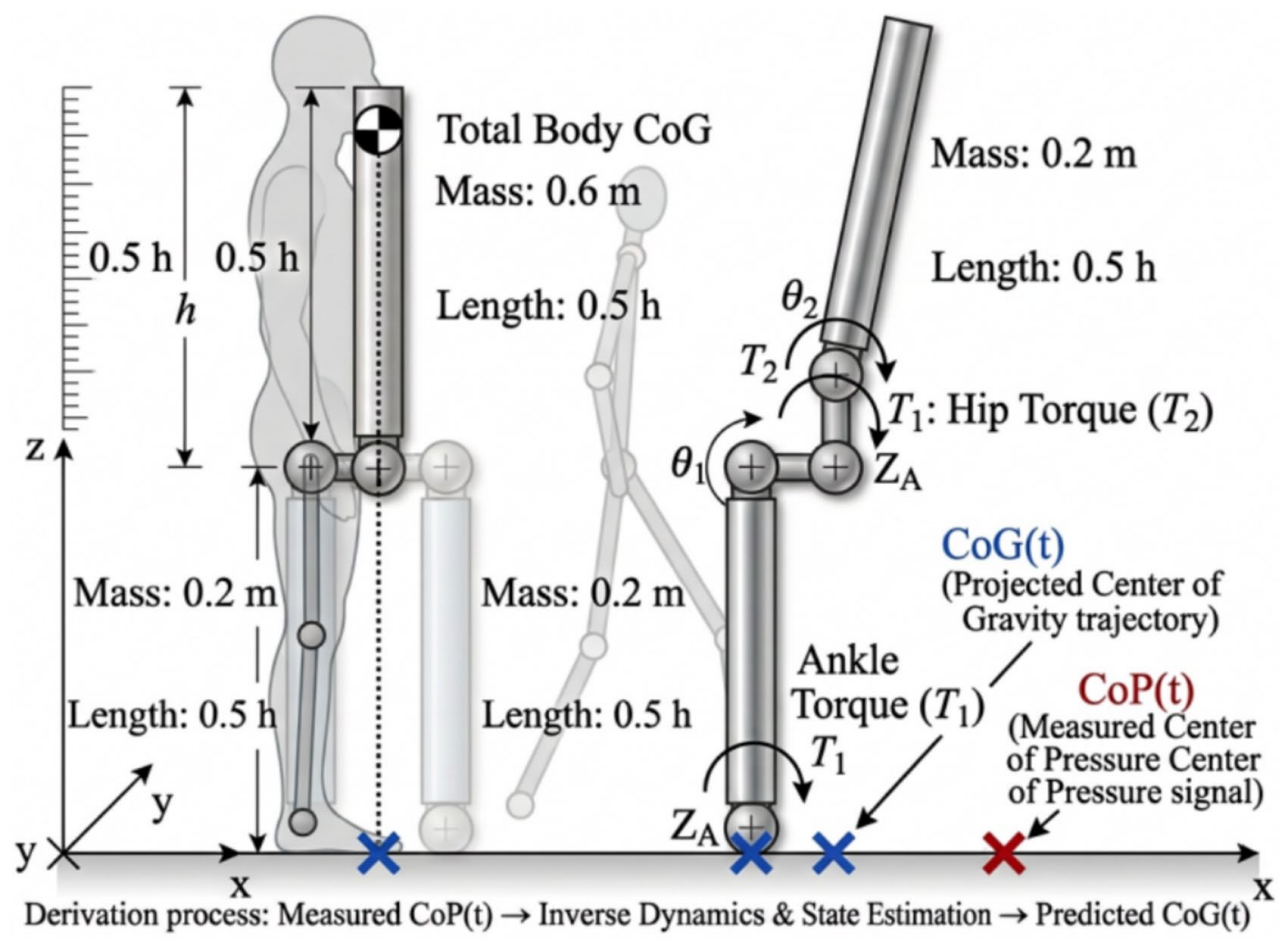
Simplified biomechanical model used to derive model-based CoG trajectories from the measured CoP signal during quiet standing.

Figure 3 documents the physical basis of this calibration procedure and the correspondence between the effective sensing area and the standardized image size used for trajectory analysis. We further derived center-of-gravity (CoG) estimates based on a human-body dynamics model driven by the measured CoP trajectory (Figure 4). In the revised manuscript, these CoG-related variables are described as model-derived estimates intended to complement the directly measured CoP descriptors, rather than as direct physical measurements of whole-body center of gravity. This clarification is consistent with prior postural-control research showing that CoP and CoM/CoG describe different but complementary aspects of standing balance control (17). While CoP reflects the net control action applied at the support surface, CoM-related motion has also been widely used to characterize balance regulation during quiet standing, and practical approaches for estimating CoM-related motion from force-platform-associated measurements have also been reported (18–20). CoG-derived metrics (e.g., mean CoG velocity) were included as complementary indicators of postural sway to facilitate comparisons with prior work.

**Figure 5:**
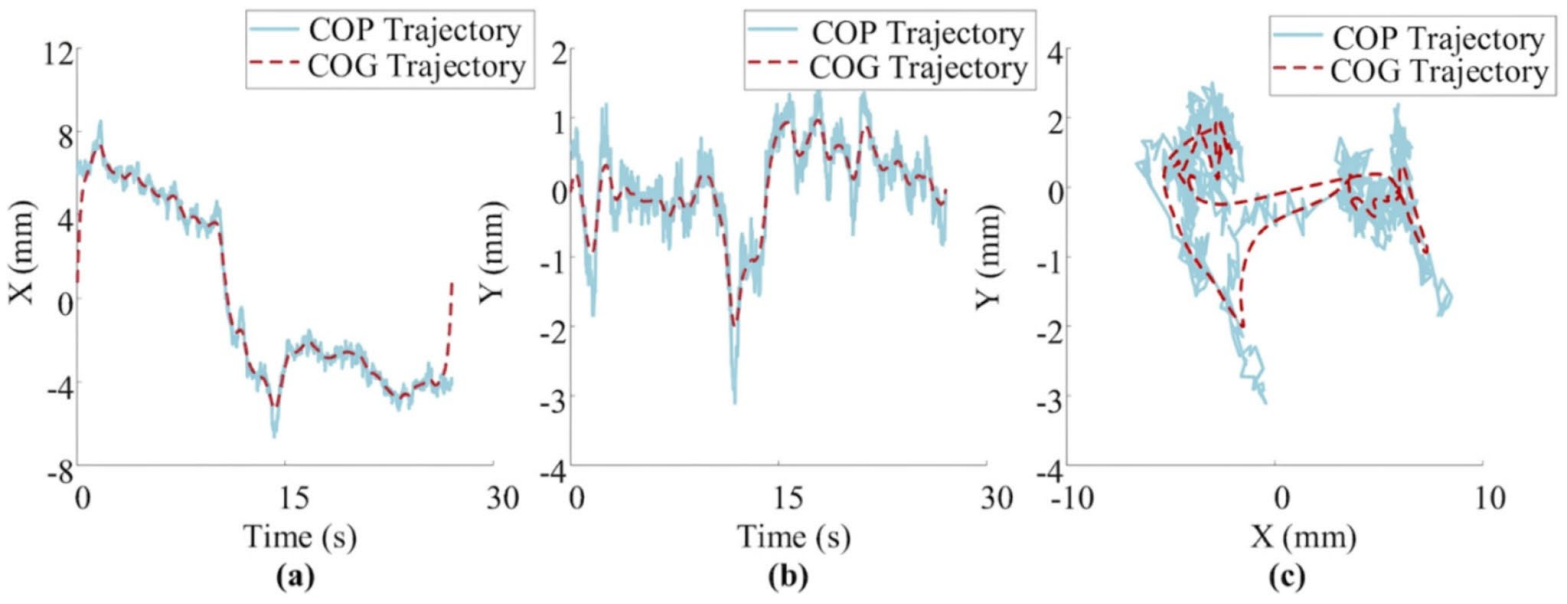
Representative CoP and CoG trajectories showing **(a)** the x-displacement time series, **(b)** the y-displacement time series, and **(c)** the two-dimensional CoP and CoG path during the standing balance test.

By combining CoP changes with this simplified model, we obtained CoG-related trajectory descriptors for subsequent analysis. The use of simplified biomechanical or inverted-pendulum models to estimate mass-center motion from force-platform-related measurements has been reported previously (18–20). However, the resulting CoG trajectory should be interpreted cautiously because it depends on simplifying biomechanical assumptions and may introduce estimation error (19). Representative CoP and model-derived CoG trajectories are shown in Figure 5.

During feature extraction, we employed a comprehensive framework spanning five domains to capture the multifaceted characteristics of balance control. Spatial-domain features quantified movement patterns by calculating the elliptical area enclosed by the CoP and model-derived CoG trajectories and the excursion range in both the anteroposterior and mediolateral directions. For stability assessment (21), we computed the root mean square (RMS) of CoP and model-derived CoG displacement and sample entropy to characterize sway magnitude as well as signal irregularity and complexity. Entropy-based measures derived from postural sway signals have been used to quantify the regularity and complexity of standing balance behavior beyond conventional linear sway metrics (22). To describe the dynamic aspects of postural control, we extracted frequency-domain features, including dominant frequency components and band-power distribution. Temporal-domain features included total path length, mean velocity, and acceleration profiles of CoP and model-derived CoG movement, reflecting the time-varying nature of balance control. In addition, we computed 63 statistical descriptors across the extracted signals, ranging from basic measures (e.g., mean and median) to dispersion and distribution metrics [e.g., standard deviation, extrema, and interquartile range (IQR)]. Collectively, these feature sets capture a broad spectrum of postural control strategies across participants.

### 2.5 Classification and model selection

**Figure 6:**
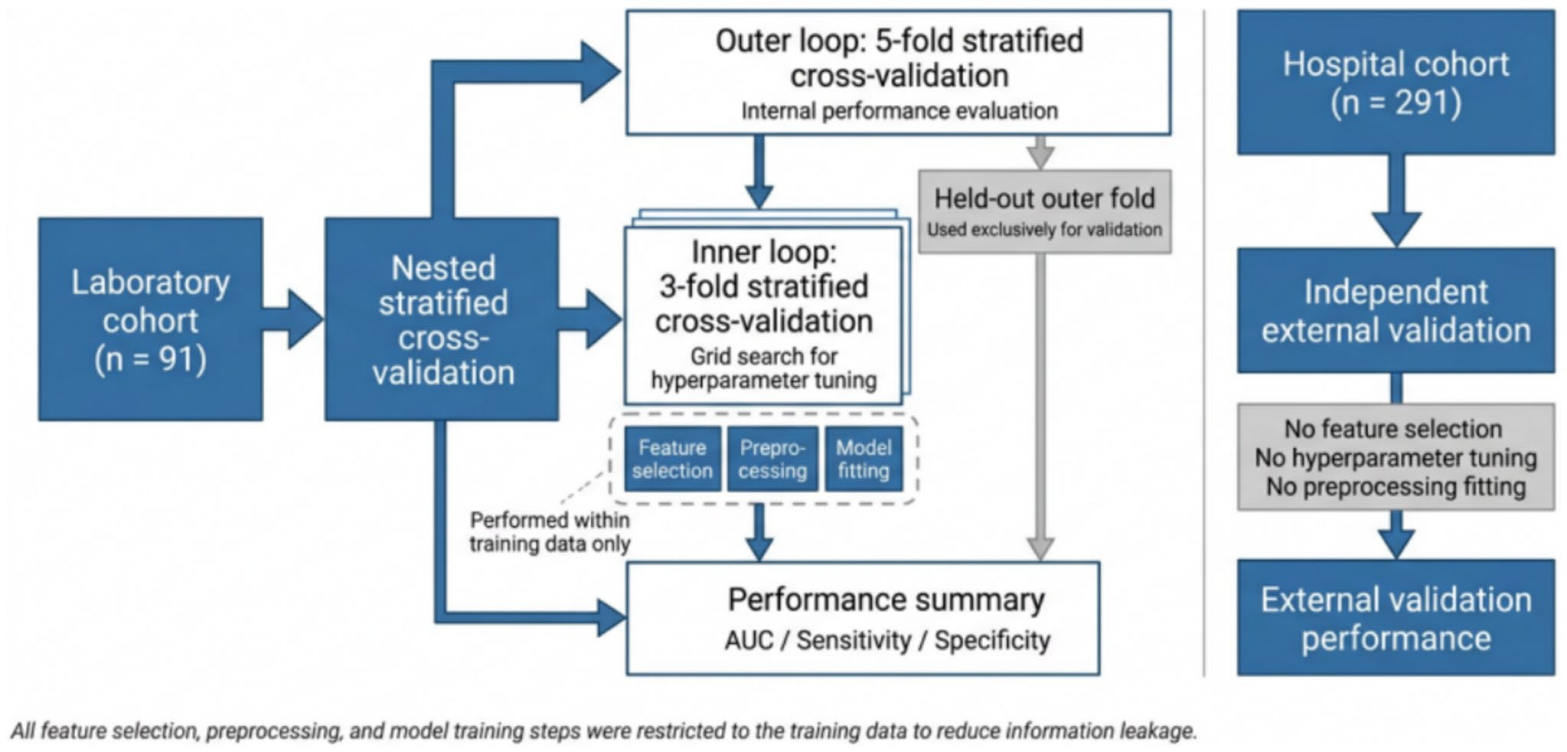
Model development and validation workflow. The laboratory cohort (*n* = 91) was used for model development under a nested stratified cross-validation framework. The outer loop consisted of 5-fold stratified cross-validation for internal performance evaluation, and the inner loop consisted of 3-fold stratified cross-validation for hyperparameter tuning by grid search. Feature selection, preprocessing, and model fitting were performed using training data only, and the held-out outer fold was used exclusively for validation. The final pipeline developed in the laboratory cohort was then applied directly to the independent hospital cohort (*n* = 291) for external validation without additional feature selection, preprocessing fitting, hyperparameter tuning, or threshold adjustment.

The primary classification analysis was conducted in the 91-participant laboratory cohort using retrospective fall status as the outcome. To improve methodological transparency and reduce the risk of optimistic bias, we restructured the revised analysis so that model development and model evaluation were clearly separated. For internal model development, we used a nested stratified cross-validation framework in the laboratory cohort (Figure 6). The outer loop consisted of 5-fold stratified cross-validation and was used for internal performance evaluation. Within each outer-loop training split, an inner 3-fold stratified cross-validation was used for hyper parameter tuning by grid search. Feature selection, preprocessing, and model fitting were all carried out only within the corresponding training data in each resampling step to reduce the possibility of information leakage. The held-out outer fold was used exclusively for validation. Performance was summarized across the held-out outer folds using AUC, sensitivity, and specificity.

We implemented and evaluated five distinct machine learning algorithms for our classification task. These included traditional statistical approaches like Logistic Regression (LR) and Naive Bayes (NB), tree-based methods such as Decision Tree (DT) and Random Forest (RF), and Support Vector Machines (SVM). To examine whether classification performance was sensitive to feature-space complexity, we compared the full extracted feature set with reduced feature subsets in supplementary model-development analyses. Because previous studies have shown that CoP and CoM-related measures provide complementary information about balance regulation (17), both directly measured CoP features and model-derived CoG-related features were retained in the candidate feature pool. However, the CoG-related variables were treated as secondary model-derived descriptors rather than primary physical measurements.

The hyper parameter optimization process encompassed five machine learning models: for balanced model capacity, Support Vector Machines [C = (0.1, 1, 10), kernel = (“linear,” “rbf”)], Decision Tree [max depth = (None, 10, 20)] to control model complexity, Random Forest [n_estimators = (50, 100, 200), max depth = (None, 10, 20)] for ensemble learning optimization, Logistic Regression [C = (0.1, 1, 10)] for regularization control, and Naive Bayes with default parameters. These candidate models and parameter ranges were defined *a priori* for model development and were not modified on the basis of the external validation cohort. For external validation, the final analysis pipeline developed in the laboratory cohort was applied directly to the independent SYP Hospital cohort without refitting. No feature selection, hyper parameter tuning, preprocessing fitting, or threshold adjustment was performed using the hospital cohort.

## 3 Results

In the revised manuscript, the results are presented in a way that is more closely aligned with the actual study design and level of evidence. The laboratory-cohort analyses are interpreted as internally validated findings for retrospective fall-history classification, the cross-cohort analysis is presented as independent external validation, and the home-based component is presented as a feasibility study of repeated at-home measurement.

### 3.1 Fall-history classification results with machine learning

**Figure 7:**
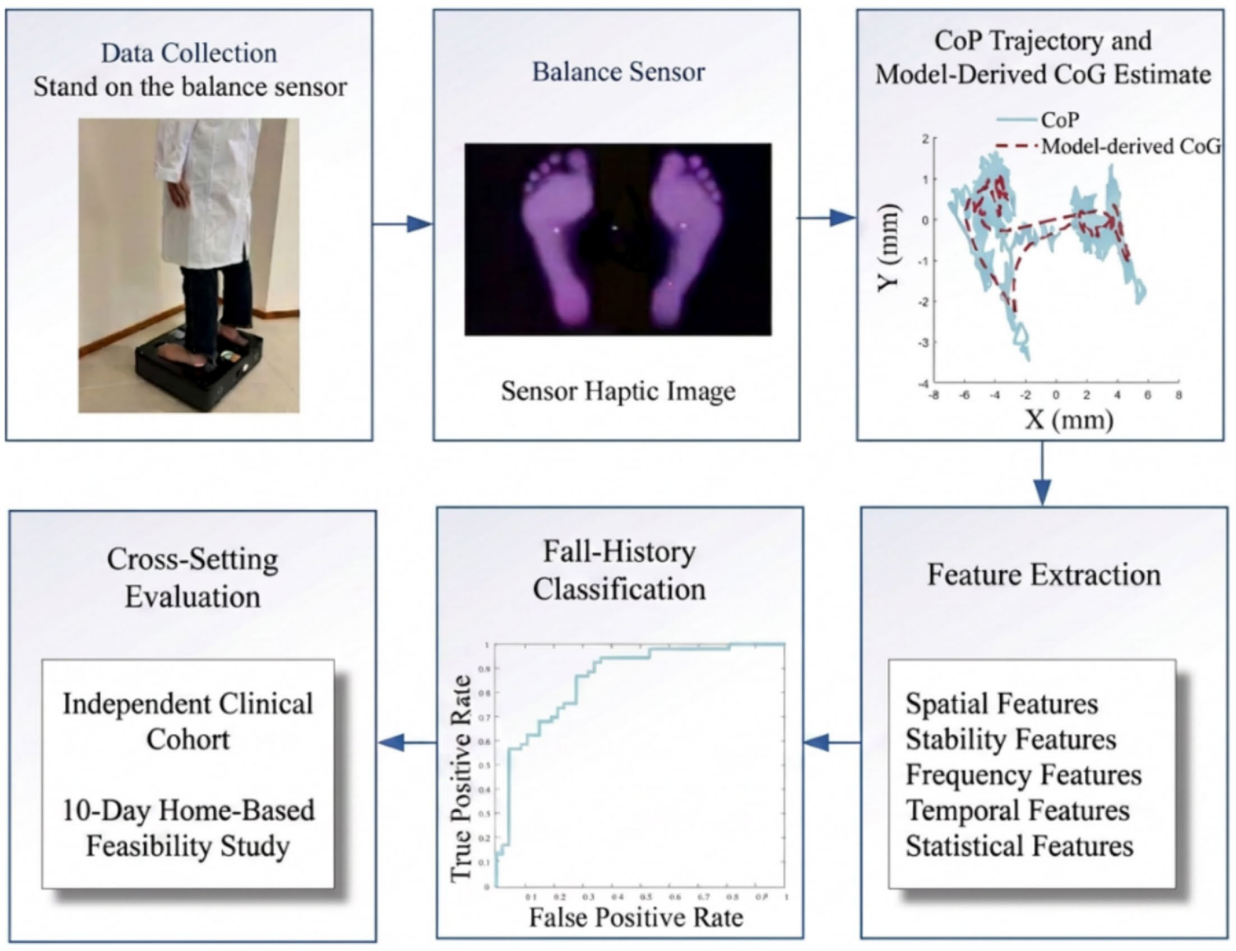
Overview of the proposed balance-sensor analysis framework. Balance data were collected while participants stood on the sensor, generating plantar-pressure haptic images. These data were used to derive CoP trajectories and model-derived CoG estimates, from which spatial, stability, frequency, temporal, and statistical features were extracted for fall-history classification. The framework was further examined through cross-setting evaluation, including an independent clinical cohort and a home-based feasibility study.

In this study, we propose a balance-sensor framework for fall-history classification and cross-setting evaluation, as illustrated in Figure 7. First, balance data were collected while participants stood on the balance sensor, generating plantar-pressure haptic images. From these data, CoP trajectories and model-derived CoG estimates were obtained to characterize postural control. Next, spatial, stability, frequency, temporal, and statistical features were extracted from the trajectories and used for fall-history classification. Finally, the framework was further examined across settings through evaluation in an independent clinical cohort and a home-based feasibility study. This workflow integrates sensor-based balance measurement, trajectory-based feature extraction, classification analysis, and cross-setting evaluation in a unified framework for fall-risk assessment in older adults.

The revised machine-learning analysis yielded more conservative performance estimates than those reported previously. In the laboratory cohort, the overall discriminative performance was moderate, with the highest AUC reaching 0.71. These results indicate that short-duration postural-sway measurements contain information relevant to retrospective fall history, although the classification performance was lower than that suggested by the earlier analysis pipeline.

**Figure 8:**
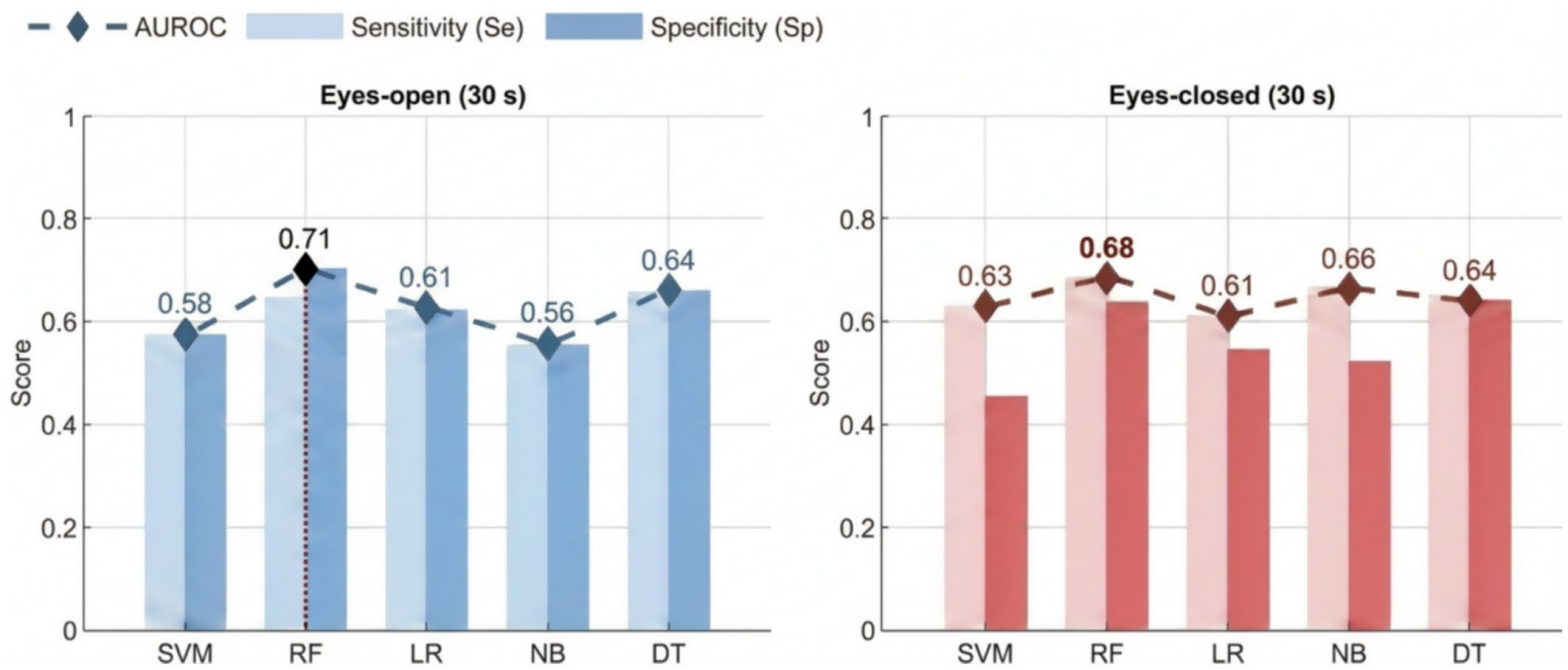
Performance comparison of machine-learning models for fall-history classification using 30-s quiet-standing balance-sensor data. Left: eyes-open; right: eyes-closed. Bars indicate sensitivity and specificity on the test set using the threshold determined from the training data; the dashed line shows AUC, with values annotated above the markers.

As shown in Figure 8, under the eyes-open (30 s) condition, Random Forest (RF) achieved the highest AUC (0.71), followed by Decision Tree (DT, 0.64), Logistic Regression (LR, 0.61), Support Vector Machine (SVM, 0.58), and Naive Bayes (NB, 0.56). Under the eyes-closed (30 s) condition, RF again showed the highest AUC (0.68), while NB, DT, SVM, and LR achieved AUCs of 0.66, 0.64, 0.63, and 0.61, respectively. Across the two visual conditions, RF showed the most favorable and relatively stable discriminative performance.

Sensitivity and specificity were computed on the held-out test set using the threshold determined from the training data. In the eyes-open condition, the RF model showed sensitivity and specificity values of approximately 0.65 and 0.70, respectively. In the eyes-closed condition, the corresponding values were approximately 0.69 and 0.64. The remaining classifiers showed lower or less balanced discrimination across conditions.

Overall, these findings support the use of balance-sensor-derived postural features for fall-history classification, while indicating a more conservative level of model performance than that suggested by the earlier analysis.

In comparison, conventional functional tests showed lower discrimination in this cohort. The Timed Up and Go (TUG) test achieved AUC = 0.60 with sensitivity = 0.65 and specificity = 0.54. The 4-m gait speed test achieved AUC = 0.51, while the sit-to-stand test achieved AUC = 0.59. Thus, the best-performing sensor-based model remained numerically higher than these conventional functional assessments.

This pattern is broadly consistent with previous reports showing that conventional single functional tests often have limited ability to identify fall-prone older adults when used in isolation. In a systematic review and meta-analysis of community-dwelling older adults, Barry et al. reported that the TUG had limited utility, with a pooled AUC of approximately 0.57 and a commonly used cutoff of 13.5 s (6). This is close to the TUG performance observed in our cohort (AUC = 0.60). Previous work has also suggested that gait speed and sit-to-stand performance alone provide only modest discrimination for falls (26, 33–35). Against this background, the higher AUC values observed for the sensor-based model suggest that pressure-derived balance features may provide complementary information beyond conventional timing-based assessments, although this interpretation remains preliminary given the sample size.

Accordingly, comparative performance is presented using the original metric values directly rather than relative percentage improvement. Table 1 and Table 2 summarize the diagnostic performance of the balance-sensor approach under the eyes-open and eyes-closed conditions, respectively, in comparison with conventional functional assessments, including Timed Up and Go (TUG), gait speed (4 m), and sit-to-stand.

**Table 1:** Comparison of sensitivity, specificity, and AUC between the balance-sensor approach under the eyes-open condition and conventional functional tests in the laboratory cohort (sample size: 91).

Measure	Balance sensor (eyes open)	Timed up and go	Gait speed (4 m)	Sit-to-stand
Sensitivity	0.65	0.65	0.50	0.54
Specificity	0.70	0.54	0.51	0.64
AUC	0.71	0.60	0.51	0.59

**Table 2:** Comparison of sensitivity, specificity, and AUC between the balance-sensor approach under the eyes-closed condition and conventional functional tests in the laboratory cohort (sample size: 91).

Measure	Balance sensor (eyes closed)	Timed up and go	Gait speed (4 m)	Sit-to-stand
Sensitivity	0.69	0.65	0.50	0.54
Specificity	0.64	0.54	0.51	0.64
AUC	0.68	0.60	0.51	0.59

Taken together with prior reports on conventional tests in older adults, these results suggest that short-duration postural sway patterns captured by the sensor contain discriminative information for fall-history classification beyond that provided by conventional functional tests alone.

### 3.2 Validation of classification method in clinical setting

#### 3.2.1 Comparison with traditional clinical assessment methods

**Figure 9:**
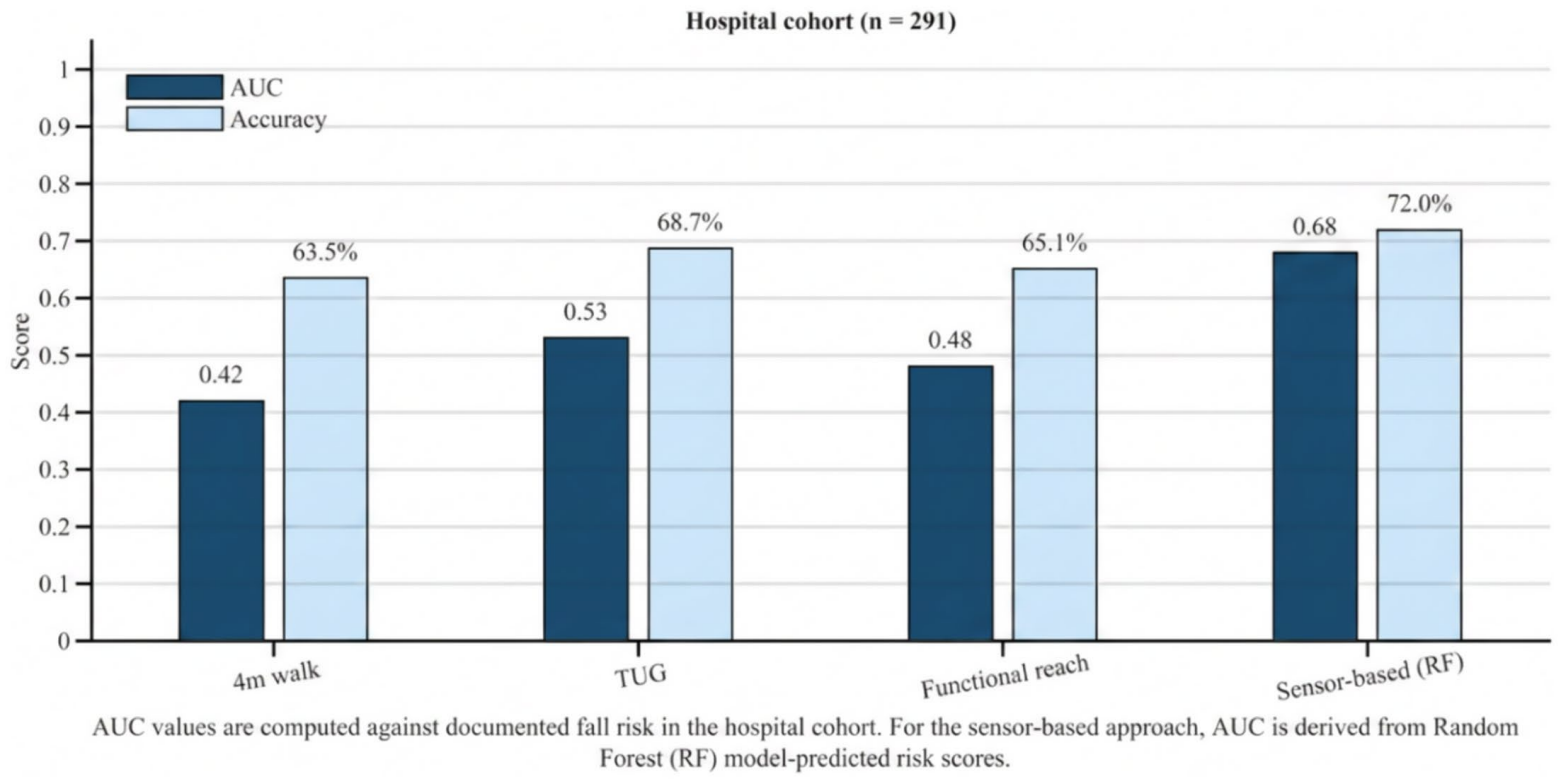
External validation performance in the hospital cohort (*n* = 291).

Figure 9 presents the external validation results of the sensor-based fall-risk model in an independent hospital cohort from SYP Hospital (*n* = 291). In the revised analysis, the laboratory cohort (*n* = 91) was used only for model development, including preprocessing, feature selection, and model training, whereas the hospital cohort was used only as an independent external validation cohort. No feature selection, hyperparameter tuning, preprocessing fitting, or threshold adjustment was performed on the hospital cohort.

Under this external-validation framework, the sensor-based model achieved an AUC of 0.68 and an accuracy of 72% in the hospital cohort. Although these values were lower than those reported previously, they provide a more conservative estimate of model performance under strict external validation.

In contrast, traditional clinical tests showed lower discrimination in the same cohort, including the 4-m walk test (AUC = 0.42), Timed Up and Go (TUG; AUC = 0.53), and Functional Reach Test (AUC = 0.48). Overall, the sensor-based model remained numerically superior to these routine clinical assessments in terms of AUC.

#### 3.2.2 Clinical implications

The revised external validation results provide a more conservative estimate of model generalizability across cohorts. Compared with conventional functional assessments, the sensor-based approach may capture additional balance-related information relevant to fall-risk classification, particularly aspects of postural control that are not fully reflected by routine clinical tests alone.

However, these findings do not establish prospective prediction of future falls, because the hospital outcome was based on documented clinical fall risk rather than longitudinally observed events. In addition, validation was performed in a single hospital cohort, and further multi-center studies will be required to evaluate robustness, transportability, and potential clinical utility in broader care settings.

The external validation results indicate that the proposed approach retains moderate discriminative performance when applied outside the original laboratory cohort. At the same time, the revised performance estimate is lower than that reported previously, underscoring the importance of strict separation between model development and model evaluation.

## 4 Discussion

This study examined whether a short-duration, sensor-based quiet-standing assessment could support fall-risk assessment in older adults using pressure-derived postural-sway features and machine-learning models. In the revised analysis, the laboratory cohort was used for internal model development and evaluation with retrospective fall history over the previous 12 months as the main outcome, whereas the hospital cohort was used as an independent external validation cohort. Under these revised and stricter analytical conditions, the sensor-based approach still showed discriminatory ability beyond that of the conventional functional tests evaluated in this study, although the performance estimates were lower than those reported in the original manuscript.

A notable implication of these findings is that a brief standing test may capture balance-related information that is not fully represented by routine functional assessments alone. Compared with tests such as Timed Up and Go, gait speed, or sit-to-stand, the balance-sensor recordings provide continuous postural-sway signals from which multiple complementary descriptors can be derived, including spatial, temporal, frequency-domain, and statistical characteristics. Rather than relying on a single summary variable, the proposed framework integrates features from several domains to characterize postural control in greater detail. This may partly explain why the sensor-based approach showed higher AUC values than the conventional assessments in both the laboratory and hospital cohorts (23–32).

The revised results should nevertheless be interpreted more cautiously than in the original submission. In response to the reviewer’s concern regarding optimistic bias and possible overfitting, we revised the analytical workflow to make the separation between model development and model evaluation more explicit. The laboratory-cohort analysis is now presented as an internally validated exploratory analysis, and the hospital-cohort analysis was performed using a fully separated external-validation pipeline. Under this stricter framework, the external-validation performance decreased compared with that originally reported. We believe that these revised estimates provide a more realistic and methodologically more appropriate indication of cross-cohort generalizability.

It is also important to clarify the interpretation of the study outcome. The main laboratory analysis was based on retrospective fall history rather than prospectively observed future falls. Accordingly, the present findings support the ability of the sensor-based method to distinguish participants according to prior fall status, but they do not by themselves establish prospective predictive validity. Similarly, the hospital-cohort analysis reflects agreement with documented clinical fall risk rather than prospective fall occurrence. We therefore revised the terminology throughout the manuscript to avoid overstating the present evidence as direct prediction of future falls.

The comparison with conventional functional tests is also worth noting. In both cohorts, the sensor-based approach showed higher AUC values than the routine assessments included in this study. One possible reason is that functional tests such as gait speed or Timed Up and Go provide relatively coarse summary measures of mobility, whereas the balance sensor captures continuous sway behavior during quiet standing. These signals may contain subtle information on postural regulation that is not readily reflected by completion time alone. From this perspective, the proposed method may serve as a useful complement to existing screening tools rather than a replacement for them.

The 10-day home-based component should also be interpreted within its intended scope. Because only 12 participants were included and only two fall events were reported during follow-up, this part of the study is best viewed as a feasibility and acceptability assessment of repeated at-home balance measurement, rather than as a validation of prospective predictive performance. Even so, it provides preliminary support for the practical deployment of the sensor in non-laboratory settings and suggests that repeated short-duration measurements may be feasible for future longitudinal monitoring studies.

Several limitations should be acknowledged. First, the laboratory cohort remained relatively small for machine-learning analysis, especially in relation to the dimensionality of the extracted feature space. Although the revised validation framework was designed to reduce optimistic bias, uncertainty remains substantial and model stability is still limited by sample size. Second, the external validation was conducted in a single hospital cohort, and broader transportability across institutions, populations, and care settings remains unknown. Third, the CoG-related variables used in this study were model-derived estimates based on a simplified biomechanical formulation driven by CoP trajectories. These variables were included as supplementary descriptors rather than as validated physical measurements, and their interpretation should therefore remain cautious. Fourth, the outcome definitions used in the present study were based on retrospective fall history or documented fall risk, not on prospectively adjudicated future-fall events. Larger prospective cohort studies with standardized outcome ascertainment will be required to determine whether the model-derived risk scores have true predictive value for future falls.

Despite these limitations, the present study suggests that a rapid, sensor-based quiet-standing assessment may provide a practical complement to existing fall-risk screening approaches. The device is non-invasive, requires only a short measurement duration, and may be suitable for repeated use in clinical, community, or home settings. Future work should focus on prospective longitudinal validation, multi-center testing, evaluation of calibration and decision thresholds, and assessment of whether the sensor-based risk score adds clinically useful information when combined with established functional and clinical assessments.

## Data Availability

The raw data supporting the conclusions of this article will be made available by the authors, without undue reservation.
